# Sanitation and Religion in South Asia: What Accounts for Differences across Countries?

**DOI:** 10.1080/00220388.2018.1469742

**Published:** 2018-05-18

**Authors:** Sangita Vyas, Dean Spears

**Affiliations:** Department of Economics, University of Texas at Austin, Austin, USA

## Abstract

Exposure to open defecation has serious consequences for child mortality, health, and human capital development. South Asia has the highest rates of open defecation worldwide, and although the incidence declines as household income rises, differences across South Asian countries are not explained by differences in per capita income. The rate of open defecation in sub-national regions of Bangladesh, India and Nepal is highly correlated with the fraction of the population that identifies as Hindu, in part because certain rituals of purity and pollution discourage having latrines in close proximity to one’s home. Almost all open defecation occurs in rural areas, and this paper estimates how much the rate could be reduced if rural households in regions that have a higher fraction of Hindus, where open defecation is still common, altered their behaviour to reflect that of non-Hindu households in regions that are predominantly non-Hindu, where the rate of open defecation is much lower. Using nonparametric reweighting methods, this paper projects that rural open defecation in Bangladesh, India, and Nepal could be reduced to rates of between 6 and 8 per cent, compared to the prevailing level of 65 per cent.

## 1. Introduction

Open defecation has important consequences for child health and human capital development. It facilitates the transmission of infectious diseases which can affect survival, physical growth, cognitive development, and adult economic productivity.^1^ Whilst open defecation has steadily been decreasing across the world, it remains notably high in rural South Asia: 60 per cent of people who defecate in the open globally live in rural South Asia, and 56 per cent live in rural India alone (WHO and UNICEF, 2015).

This study considers associations between religion and latrine use, and is the first to examine the relationship at a multi-country level. Rural South Asia represents 60 per cent of the global open defecation burden, and 93 per cent of the region’s burden, and is the focus of this paper. The two countries with the highest rates of rural open defecation in the region, India and Nepal, are also the countries with the highest fraction of Hindus. A cultural reason for this is that certain practices in Hinduism influence sanitation through strict observance of rituals of purity and pollution that mean many Hindus in rural areas are resistant to having latrines in close proximity to their homes. These aspects of religion are discussed in Section 2.

This paper makes three contributions to the literature. It is the first we are aware of to document the aggregate correlation between Hinduism and open defecation in South Asian countries, thus advancing a growing body of evidence linking latrine adoption to culture and religion. Second, it contributes to the economic and demographic literatures linking religion to behavioural outcomes. Finally, it uses demographic reweighting methods, a standard empirical strategy in the literature, to project counterfactual rates of rural open defecation if households in regions with a higher fraction of Hindus demonstrated the same sanitation behaviours as non-Hindu households in the best performing largely non-Hindu regions.

A large body of research in demography considers aggregate consequences of religion for demographic outcomes. In the United States, the role of religion for outcomes such as marriage, fertility, women’s labour force participation, education, and wages has been widely documented (Lehrer, 2004). In the context of India, there has been considerable discussion among demographers on the role of religion in explaining aggregate differences in fertility and child mortality between Hindus and Muslims (Dharmalingam & Philip Morgan, 2004; Guillot & Allendorf, 2010; Jeffery & Jeffery, 2002; Philip, Stash, Smith, & Mason, 2002). Dyson and Moore (1983) highlight the importance of religion to understanding differences in demographic outcomes in certain contexts, for instance between Bangladesh and West Bengal, and between Punjab and other north Indian states. Our study contributes to this literature by utilising demographic methods to understand the links in South Asia between religion and sanitation, which is an important input globally to demographic change and declining early-life mortality.

Research on latrine adoption in South Asia has primarily focused on economic constraints, infrastructure, and social influence (Duflo, Greenstone, Guiteras, & Clasen, 2015; Guiteras, Levinsohn, & Mobarak, 2015; O’Reilly & Louis, 2014; Pattanayak et al., 2009). While Banda et al. (2007) identify the importance of socio-cultural factors for latrine adoption in rural India, only very recently have researchers begun to investigate religion in particular. Geruso and Spears (2018) study differences in infant mortality rates between Indian Hindus and Muslims, and present evidence that the higher mortality rates observed among Indian Hindus can be completely accounted for by the different sanitation environments in which Hindu and Muslim children grow up. The findings of qualitative studies point to the role that religious beliefs and caste relations, which originate in Hinduism, play in sanitation behaviour (Coffey et al., 2017a; Coffey & Spears, 2017; O’Reilly, Dhanju, & Louis, 2017; Routray, Schmidt, Boisson, Clasen, & Jenkins, 2015). These studies argue that beliefs in purity and pollution contribute to the acceptability of widespread open defecation and the rejection of inexpensive latrines in rural India. Spears and Thorat (in press) investigate the role of purity and pollution quantitatively in India and present evidence that households are more likely to defecate in the open in places where a greater fraction of their neighbours practice untouchability, meaning where norms of purity and pollution are more enforced.

Section 2 discusses the religious aspects that are relevant for latrine adoption. Section 3 presents summary statistics and region-level analyses. Section 4 introduces the two methods we use to estimate counterfactuals, nonparametric reweighting and linear probability models, and Section 5 presents the results. The final section discusses the implications for research and policy.

## 2. Background and context

It is not the aim of this paper to explain the origin of sanitation differences between Hindus and non-Hindus, and it is possible that these differences arose because of some unobserved factor also associated with religion. It is also the case that Hindus and non-Hindus are both heterogeneous groups with substantial variation in sanitation behaviour: many Hindus do use latrines regularly, and many non-Hindus do not (Appendix Figure A2 in Supplementary Materials illustrates this point graphically).

Anthropologists have long discussed the importance of Hindu beliefs in purity and pollution in relation to the caste system and sanitation behaviour (Srinivas, 1952). In Indian society, castes that are considered to be higher ranking are believed to embody greater purity than castes considered to be lower in rank. People from lower castes have traditionally been compelled to perform degrading tasks that require contact with ritually polluting substances, such as faeces, and doing this work has been used as evidence of their permanent pollution and justification for their social exclusion. For generations, and still today, sanitation in India has been intricately linked with the caste system. More than a century ago, in order to challenge the hierarchies of the caste system, Gandhi urged his followers to clean the excreta from their own primitive latrines. More recently, scholars in India attribute the persistence of open defecation and poor sewage management to the persistence of the caste system (Gatade, 2015; Ramaswamy, 2005; Teltumbde, 2014).

The context presented by Hindu notions of purity and pollution and the caste system has important implications for latrines and latrine use in rural India and Nepal. Through qualitative interviews, Coffey et al. (2017a), O’Reilly et al. (2017), and Routray et al. (2015) find that rural households in India and the Nepali terai believe the accumulation of faeces in a latrine near the home threatens the purity of the home. Specifically, Coffey et al. (2017a) find that the types of latrines that most rural households could afford, with pits that fill up every five years or so, are considered to be particularly problematic because they require periodic emptying. Manually emptying a latrine pit is an unpleasant job anywhere, but in India and Nepal, it is particularly fraught because of its association with work that people from castes considered to be lower ranking have traditionally been forced to do. People from castes considered to be higher in rank would not want to empty a latrine pit for fear of ritual pollution, and the social stigma associated with it. At the same time, caste is slowly being renegotiated in these societies and those from lower castes are understandably abandoning the ritually impure jobs associated with their oppression. Because of limited labour supply and a slow shift in social attitudes, finding someone to manually empty a latrine pit in rural India and Nepal can be difficult, expensive, and socially uncomfortable. O’Reilly et al. (2017) argue that households from castes considered to be lower ranking are similarly reluctant to use latrines since latrine pit cleaning reinforces caste hierarchies. To avoid the challenges brought about by having and using a latrine, most prefer not to use a latrine at all, while some very rich households build and use one that has a very large septic tank that is only emptied occasionally by a truck that mechanically vacuums out the contents. Although septic tanks help circumvent concerns around purity and pit emptying, they are prohibitively expensive to construct for the average rural household.

### 2.1. Data and the paradox of sanitation in South Asia

We primarily use household-level data from Bangladesh, India, and Nepal’s nationally representative Demographic and Health Surveys (DHS). The DHS from the remaining South Asian countries either do not contain data on religion, or the last available survey is from before 1990. Appendix Table A1 (in Supplementary Materials) describes data availability in the DHS surveys we use. We also use country-level data on open defecation, the fraction Hindu, and other covariates from various sources including the WHO and UNICEF Joint Monitoring Programme for Water Supply and Sanitation (JMP), World Bank World Development Indicators, and country censuses.

The outcome variable of interest in this analysis is household open defecation. In the DHS, each household is classified as defecating in the open or not according to its report of where members ‘usually’ defecate. Our key independent variable is household religion (see Appendix A1 for details on how this variable was constructed). We construct an indicator for social norms by calculating the fraction of households that report identifying with a religion other than Hinduism. This indicator is calculated at the division level in Bangladesh, at the state level in India, and at the region level in Nepal. Appendix Table A2 (in Supplementary Materials) lists the subnational levels and associated summary statistics for the three countries. For simplicity, in the remainder of this paper, we refer to ‘divisions, states, and regions’ simply as ‘states’. In addition, we use DHS data on literacy (ability to read or write), educational attainment (number of years of education), demographic controls for the number of household members in different age by sex bins, piped water supply in the dwelling or plot, electricity access, and binary indicators for ownership of a radio, television, refrigerator, bicycle, motorcycle, mobile, table, chair, and fan.

Open defecation rates vary widely across the countries of South Asia, but the differences present a puzzle that is not solved by standard indicators of development. Table 1 compares South Asian countries on various indicators including open defecation, GDP per capita, poverty, literacy, and access to electricity and improved drinking water (similar observations comparing India to sub-Saharan Africa have been made in Coffey et al., 2017a). Although globally richer countries tend to have better sanitation than poorer countries, within South Asia the countries that have the highest rates of open defecation present a paradox. Bangladesh has a per capita income that is roughly half of India’s, yet rural open defecation there is a much lower 4 per cent compared to India’s 64 per cent. Poverty does not appear to account for differences across countries either. Of the available data, the fraction of the population living under $3.10 per day is the highest in Bangladesh, which has the third lowest rate of rural open defecation, after the Maldives and Sri Lanka, where no one defecates in the open. Illiteracy, electricity coverage, and access to improved drinking water, have similarly little to do with differences in rural open defecation rates in the region.

**Table 1.** Summary statistics: development indicators and open defecation in South Asia

indicator	rural open defecation	fraction Hindu	rural improved water access	rural population (millions)	IMR	GDPpc	rural electricity	poverty, under $3.10/day	female literacy
source	JMP, 2012	latest Census	JMP, 2012	– – – World Bank, 2012 – – –				World Bank, multiple year	
(1)	(2)	(3)	(4)	(5)	(6)	(7)	(8)	(9)	(10)
India	64%	80%	90%	863.90	43	$4,861	70%	58%	59%
Nepal	44%	81%	88%	22.68	33	$2,155	72%	48%	49%
Pakistan	28%	2%	89%	110.00	71	$4,380	91%	45%	43%
Afghanistan	19%	<1%^a^	44%	22.16	72	$1,899	32%	N/A	18%
Bhutan	4%	22%	97%	0.47	31	$7,120	53%	14%	39%
Bangladesh	4%	9%	84%	105.59	35	$2,715	49%	78%	56%
Sri Lanka	0%	13%	92%	16.61	9	$10,028	86%	14%	90%
Maldives	0%	<1%^a^	98%	0.20	9	$12,469	100%	18%	98%

*Notes*: Sources as indicated except ^a^indicates CIA World Factbook estimates (Maldives is officially Muslim); JMP is the WHO and Unicef Joint Monitoring Programme for Water Supply and Sanitation; and World Bank indicates World Bank World Development Indicators. Infant mortality rate (IMR) is per 1000 live births, and per capita GDP (GDPpc) is in 2011 international purchasing power parity $US.

## 3. Correlations between Hinduism and open defecation

**Figure 1.**
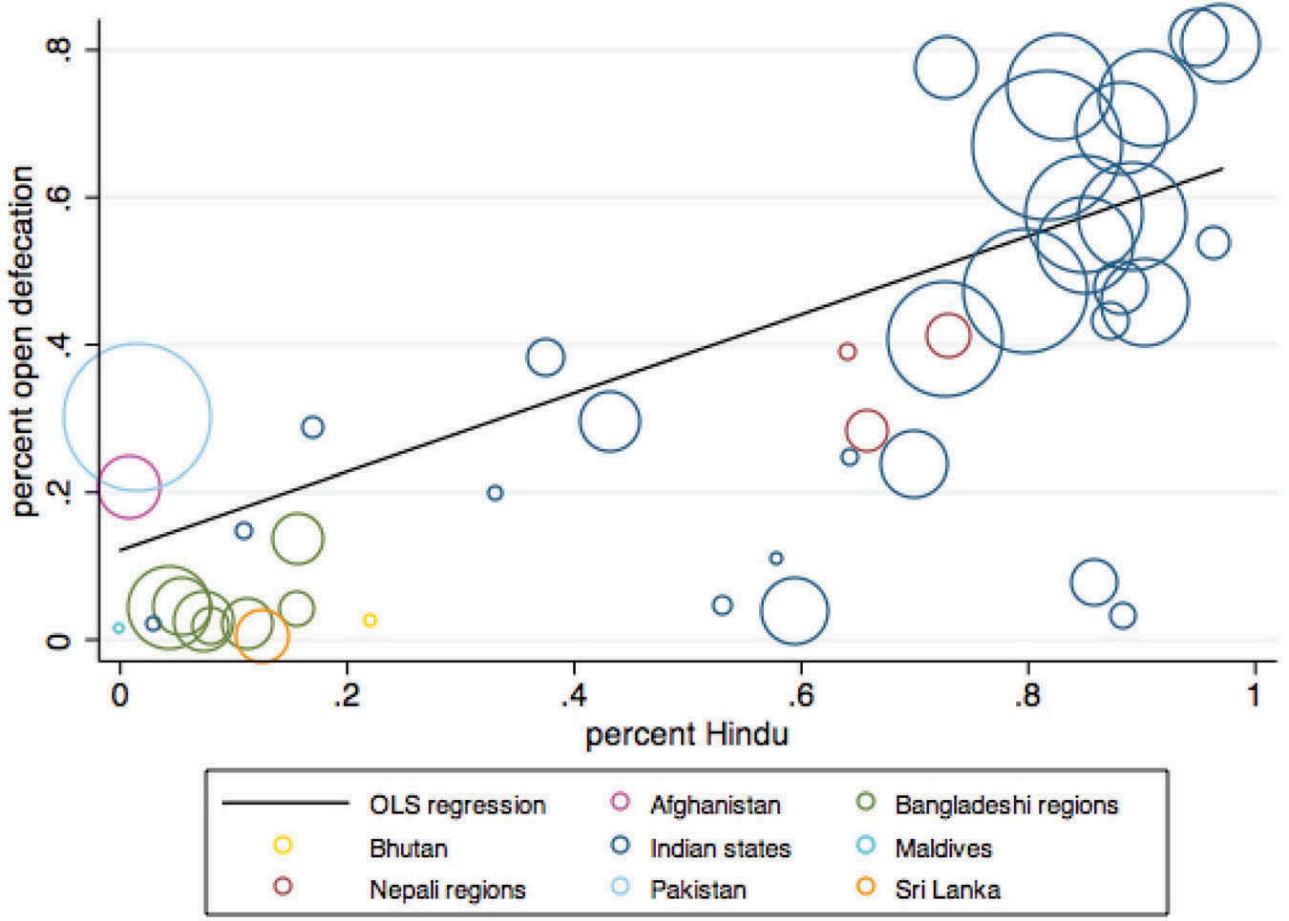
Hinduism and open defecation across parts of South Asia. *Notes*: Each circle represents a country or a part of a country, and the size of the circle represents total population size. Seven administrative divisions in Bangladesh, 29 Indian states, and three Nepali regions are depicted separately. Data for the remaining countries are shown at the country level. Figure plots a univariate OLS regression of the fraction of the population defecating in the open on the fraction of the Hindu population, using sampling weights.

Another difference among these countries is religion. India and Nepal, with the highest rates of rural open defecation, are predominantly Hindu, whereas the rest of the countries in South Asia, with lower rates of rural open defecation, are predominantly non-Hindu. Figure 1 depicts an initial result to motivate the subsequent main analysis of this paper: a positive correlation between Hindu fraction of the population and rural open defecation for South Asian countries. Each circle on this graph represents a state or country, and the size of the circle represents the size of the population. We use DHS data to the extent that it is available, and in its absence, we use data from Table 1. The graph plots state-level data for Bangladesh, India, and Nepal, and country-level statistics for the remaining countries in South Asia. The figure shows a strong relationship between Hinduism and open defecation: a higher fraction of Hindus is associated with greater open defecation across South Asia. Most of the Indian states are clustered near the top right, with high rates of open defecation, and a high fraction of Hindus. The Nepali regions are in the middle. The Bangladeshi divisions and the remaining countries in South Asia are clustered near the bottom left, with low rates of open defecation and majority non-Hindus.

The regression line in the figure is plotted using population weights and corresponding OLS regression results are presented in Table 2. Column 1 of Table 2 presents results from a univariate regression, and Column 2 shows that the relationship is robust to the inclusion of covariates. In both models, the coefficient on fraction Hindu is large, positive, and statistically significant. The point estimates in these regressions suggest that places that are 100 per cent Hindu have open defecation rates that are at least 50 percentage points higher on average than places that have no Hindus. Fraction Hindu alone accounts for more than half of the variation in open defecation across subnational regions and countries in South Asia, and after including covariates, the model accounts for 89 per cent of variation across places.

**Table 2.** Hinduism and open defecation across parts of South Asia

	(1)	(2)
Hindu, %	0.533***	0.575***
	(0.107)	(0.0874)
female literacy, %		−0.761***
		(0.183)
water piped to dwelling or plot, %		0.292
		(0.225)
electricity, %		0.00241
		(0.220)
radio, %		0.0219
		(0.136)
television, %		−0.603
		(0.494)
refrigerator, %		0.185
		(0.336)
bicycle, %		0.145
		(0.156)
motorcycle, %		0.110
		(0.447)
number of states or countries	44	42
R-squared	0.588	0.891

*Notes*: Estimates displayed are from OLS regressions. Robust standard errors are in parentheses, and two-sided p-values are displayed as *** p < 0.01, ** p < 0.05, * p < 0.1. Data for Bangladesh, India, and Nepal are at the subnational level, and for the rest of South Asia at the country-level. Data on covariates are not available for Bhutan and Sri Lanka.

Column 2 also provides evidence on how other factors are correlated with open defecation. Interestingly, the only statistically significant covariate is female literacy; places with higher female literacy have lower rates of open defecation. What is notable is that the magnitude of the point estimate on fraction Hindu is about as large as that of the education variable. Religion is thus an important factor in accounting for differences across places in South Asia.

**Figure 2.**
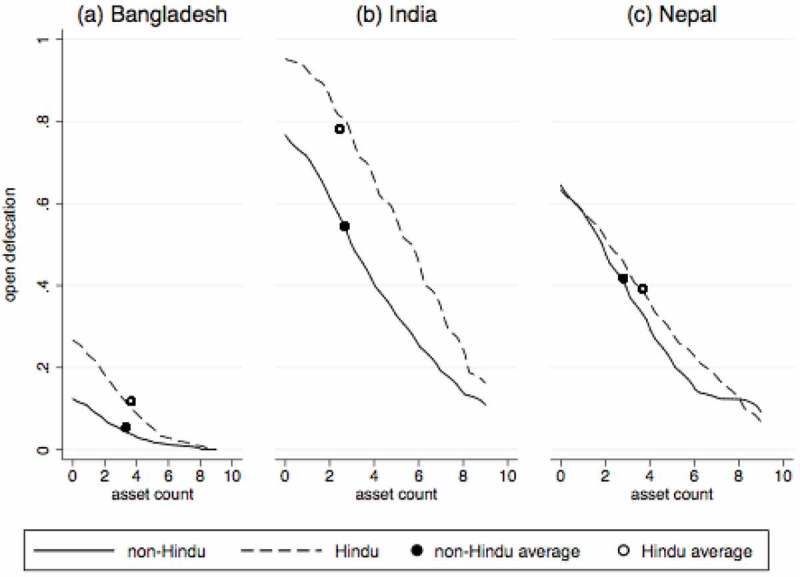
Open defecation by religion and level of asset wealth in Bangladesh, India, and Nepal. *Notes*: Each panel presents local non-parametric regressions, along with collapsed averages, by religion.

Figure 2 explores the association between religion and open defecation using household-level data for Bangladesh, India, and Nepal, the countries for which household religion data are available since 1990. This figure shows the fraction of households reporting open defecation at different levels of asset wealth in each country, separately for Hindus and non-Hindus. The asset count is a simple sum of nine assets (listed in Section 2.1). The main finding from this figure is that in each of these countries, Hindus are more likely than non-Hindus to defecate in the open at almost every level of asset wealth.

It is noteworthy that in each of these countries, the difference in open defecation rates between Hindus and non-Hindus is smaller than the difference in open defecation rates between households with zero assets versus households with nine assets. Ignoring social spillovers, this could suggest that wealth may be more important for sanitation uptake than religious beliefs, particularly in India where the slope is the largest. However, few households have as many as nine assets. In India, households in the 25th percentile of asset ownership, who own one asset, are 29 percentage points more likely to defecate in the open than households in the 75th percentile, who own four assets. Comparing this to the overall average difference in open defecation between Hindus and non-Hindus of 23 percentage points suggests that religion is at least as important a factor for sanitation behaviour as wealth is.^2^ See Supplementary Materials Figure A1 for the cumulative distribution of asset ownership.

Finally, the differences in rates of open defecation among Hindus across these three countries suggest that social norms are important for sanitation behaviour. In Bangladesh, 90 per cent of the population is Muslim, while in India and Nepal, at least 80 per cent are Hindu. Only 12 per cent of rural Hindus defecate in the open in Bangladesh, as opposed to 78 per cent in India. Another fact that supports the presence of social spillovers is that in rural Bangladesh, India, and Nepal, 35 per cent of Hindu households and 10 per cent of non-Hindu households defecate in the open in states that are at least 50 per cent non-Hindu, whereas 77 per cent of Hindu households and 57 per cent of non-Hindu households do so in states that are less than 50 per cent non-Hindu.

**Figure 3.**
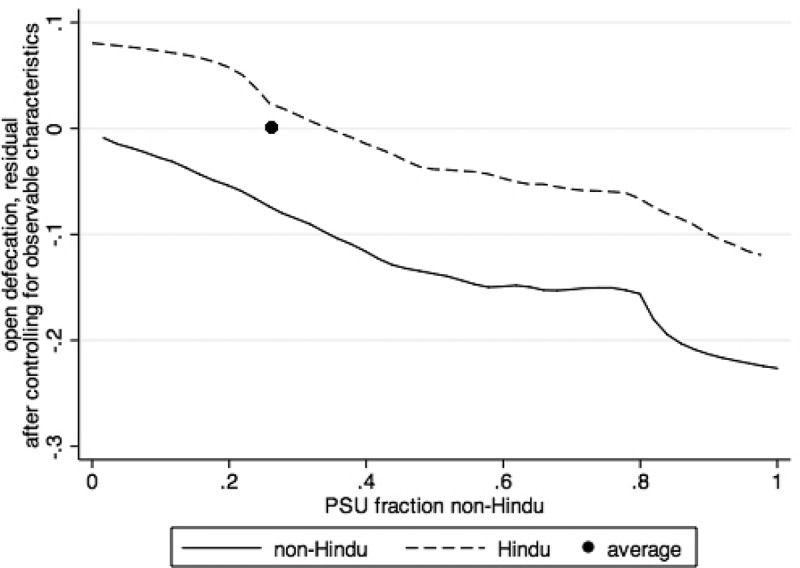
Social spillovers in sanitation behaviour for rural Hindu and non-Hindu households in Bangladesh, India, and Nepal. *Notes*: Figure presents local non-parametric regressions by religion, along with a collapsed average. Controls include household ownership of nine assets, years of education of the most educated member of the household, electricity access, piped water, and 10 demographic controls for the number of household members in different age by sex ranges.

Figure 3 explores the evidence on social spillovers graphically. After controlling for important covariates, both Hindus and non-Hindus are less likely to defecate in the open if they live in primary sampling units (PSUs), or villages, with a higher fraction of non-Hindus, and are more likely to defecate in the open if they live in PSUs with a higher fraction of Hindus. Not only are non-Hindus themselves more likely to use a latrine, but the greater prevalence of non-Hindus within a community is associated with less open defecation among households of all religions. Conversely, the norms and beliefs rooted in Hinduism not only influence the behaviour of Hindus, but also the behaviour of non-Hindus living in areas where these beliefs are more prevalent.

## 4. Methods

The data show that Hinduism is correlated with open defecation. However, this behaviour is not fixed: open defecation rates are relatively lower for Hindu households living in areas with larger non-Hindu populations, where social norms are more likely to promote latrine use. What would the level of rural open defecation in Bangladesh, India, and Nepal be if sanitation norms across the region reflected the norms in the areas with the lowest open defecation? We explore this question by estimating a rural rate of open defecation for Bangladesh, India, and Nepal, if households in regions that have a higher fraction of Hindus, where open defecation is common, demonstrated the same sanitation behaviours as non-Hindu households in largely non-Hindu regions, which have the lowest levels of open defecation. We use two complementary strategies: a nonparametric reweighting strategy of the sort pioneered by DiNardo, Fortin, and Lemieux (1996),^3^ and linear probability models.

### 4.1. Method one: nonparametric reweighting

A simple projection would apply the average rate of open defecation across all non-Hindu households to the entire Hindu population. However, Hindu and non-Hindu households in these countries differ. Non-Hindu households are less likely to defecate in the open, but Table 3 shows that they also have more assets, are less educated, and are less likely to have infrastructure like electricity and piped water, on average. These factors are believed to be linked to latrine adoption (Duflo et al., 2015; Jenkins & Curtis, 2005; O’Connell, 2014; O’Reilly & Louis, 2014; Routray et al., 2015), and thus should be controlled for in some way.

**Table 3.** Differences between latrine users and non-users, and between Hindus and non-Hindus in rural Bangladesh, India, and Nepal

	household uses latrine	household defecates in the open	Hindu	non-Hindu
	(1)	(2)	(3)	(4)
non-Hindu	0.504	0.134	–	–
fraction non-Hindu in state	0.476	0.189	0.193	0.562
asset count (of 9)	4.004	1.874	2.499	2.986
years of education	8.739	6.174	7.253	6.609
electricity	0.686	0.482	0.568	0.515
piped water	0.150	0.0830	0.118	0.0740
defecates in the open	–	–	0.761	0.327

*Notes*: Means calculated using sampling weights.

To accommodate for these differences, we partition our sample into groups based on observable characteristics, including number of assets owned, educational attainment, and having electricity and piped water. We then apply the mean rate of open defecation among the non-Hindus in each group to the Hindus in the same group. In other words, we reweight non-Hindu households to match the distribution of our set of observable characteristics for Hindu households, thereby constructing a counterfactual mean rate of open defecation among Hindus.

We construct a reweighting function, Ψ(*y*), such that

(1) $$\Psi \left(y\right)\times f\left(y|R=NH\right)=f\left(y|R=H\right),$$

where *f* is a probability density function, *y* is a vector of observable characteristics correlated with open defecation, and *R* denotes religion. This function allows us to change the distribution of a set of observable characteristics for non-Hindu households so that it matches the distribution for Hindu households. For example, if asset ownership is the only observable characteristic considered, for each *x* number of assets owned, we calculate our reweighting function by dividing the fraction of Hindu households owning *x* assets by the fraction of non-Hindu households owning *x* assets. These new weights make the asset distribution for our non-Hindu population look like the asset distribution for our Hindu population. By calculating average open defecation among our reweighted non-Hindus, we effectively create a counterfactual rate of open defecation for Hindus if they exhibited the same sanitation behaviours as non-Hindus but otherwise kept the actual Hindu distribution of asset wealth.

As we saw in Figure 3, a household’s decision to construct a toilet is also influenced by the social norms and cultural meanings associated with latrine use. A randomised controlled trial in Bangladesh documented an effect of latrine subsidies on latrine ownership among both subsidised and neighbouring unsubsidised households (Guiteras et al., 2015). Qualitative research on successful sanitation adoption in India similarly highlights the importance of growing latrine adoption among neighbours to one’s own decision to build and use a latrine (O’Reilly & Louis, 2014).

If Hindu households exhibited the same sanitation behaviours as non-Hindu households, social norms surrounding latrine use would be very different. Thus, non-Hindu Indian and Nepali households that live in areas that are majority Hindu, where open defecation is more prevalent, may not be the best example of the counterfactual we wish to observe because their behaviour may be influenced by social norms promoting open defecation. A better example of the desired counterfactual may then be non-Hindus living in Bangladesh, a majority Muslim country, and certain states of India that have high fractions of non-Hindus, because these places already have stronger social norms promoting latrine use.

In practice, we restrict the sample of non-Hindu households used to project open defecation based on the fraction of non-Hindus in the household’s state. If the cutoff fraction of non-Hindus in the state is set to *x*, the households used to make projections, or the households that are reweighted, are the non-Hindu households living in states with a non-Hindu fraction of at least *x*. Because non-Hindu households may behave differently if they were to live in even more non-Hindu neighbourhoods, we create counterfactual open defecation rates for Hindus and for the remaining sample of non-Hindus, those living in states with a fraction of non-Hindus lower than *x*. Thus, we reweight the non-Hindu households living in areas that are at least *x* per cent non-Hindu to match the characteristics of all Hindu households, and also those of non-Hindu households living in areas less than *x* per cent non-Hindu. Practically, our method of incorporating social spillovers reweights a smaller and smaller sample to make projections for more and more households. In the most restricted scenario, we reweight rural non-Hindus living in Mizoram, the state with the highest fraction of non-Hindus of 97 per cent, to create counterfactual open defecation rates for the remaining rural households. We therefore modify our reweighting function, $\tilde{\Psi }$(*y*), as follows:

(2) $$\tilde{\Psi }\left(y\right)=\frac{f\left(y|R=H\;or\;R=\;NH\;for\;\bar{NH_{S}}\;<x\right)}{f\left(fy|R=NH\;for\;\bar{NH_{S}}\geq x\right)},$$

where the numerator includes Hindus and non-Hindus living in states that are less than *x* per cent non-Hindu, and the denominator includes only non-Hindus living in states that are at least *x* per cent non-Hindu. Thus, the counterfactual rate of rural open defecation is calculated as follows:

(3) $$\bar{OD}=\frac{n^{H}+n^{NH\;for\;\bar{NH_{S}}\;<x}}{n^{NH\;\;\bar{NH_{S}}\;\geq x}\left(n^{NH}+n^{H}\right)}\sum_{j}^{n^{NH\;for\;\bar{NH_{S}}\;\geq x}}[OD_{j}\times \tilde{\Psi }\left(y_{j}\right)]+\frac{1}{n^{NH}+n^{H}}\sum_{j}^{n^{NH\;for\;\bar{NH_{S}}\;\geq x}}OD_{j},$$

where *n^H^* is the total number of Hindu households, *n^NH^* is the total number of non-Hindu households, *j* indexes non-Hindu households living in states that are at least x per cent non-Hindu, and *OD* is a binary indicator for whether the household defecates in the open or not. The first part of the equation reweights non-Hindu households living in states that are at least *x* per cent non-Hindu based on the characteristics of Hindu households and non-Hindu households that live in states that are less than *x* per cent non-Hindu, and the second part of the equation simply adds in the true rate of open defecation of these same non-Hindu households indexed by *j*.

In practice, in order to reweight non-Hindus to match the distribution for Hindus, there must be at least some non-Hindu households for each specific combination of observable characteristics for which there are Hindus. In some cases, there are no non-Hindus for a given combination of observable characteristics. In these cases, we group the corresponding Hindu households with those having one fewer asset, but the same characteristics for educational attainment, electricity, and piped water.

### 4.2. Method two: linear probability models

We compare the results of the demographic reweighting method using coefficients from regression analysis. Regression analysis computes average associations, holding other covariates constant. Thus, it is less flexible than demographic reweighting because it matches Hindus and non-Hindus on observable characteristic means and assumes linearity, rather than matching over the entire joint distribution of observed characteristics over which the reweighting is calculated. However, regression analysis has the advantage that estimated marginal ‘effects’ are transparent and able to be compared under alternative specifications. Regression analysis is therefore a useful tool for testing the robustness of the results obtained through demographic reweighting.

Using data from rural Bangladesh, India, and Nepal, the regression we estimate is:

(4) $$OD_{is}=\beta_{1}household\;nonHindu_{is}+\;\beta_{2}state\;nonHindu_{s}+P_{s}\psi +A_{is}\theta +B_{is}\phi +E_{is}\varphi +\varepsilon_{is},$$

where *i* indexes households and *s* indexes states. Standard errors are clustered by survey PSU. The dependent variable, *OD*, is a binary indicator for whether members of the household ‘usually’ defecate in the open or not. The main coefficients of interest in this analysis are those on *household non-Hindu*, which is a binary indicator, and *state non-Hindu*, which is a fraction from zero to one, constructed by averaging over all households in the state, both urban and rural. We include four further sets of controls in order to demonstrate the robustness of our regression specification:

- $P_{s}$, state-level rural wealth: state-level average of rural household ownership of nine assets. Asset ownership is first calculated at the household level as a simple sum over ownership of nine assets (listed in Section 2.1). An average of the asset ownership variable is calculated by state, using only rural households. This serves as a control for state-level differences in rural development.
- $A_{is}$, assets and education: a simple sum of assets owned and highest level of education among household members. These variables serve as controls for household wealth and socio-economic status.
- $E_{is}$, household infrastructure: binary indicators for electricity access and water piped into the dwelling or plot. These variables serve as additional controls for household wealth, and are also believed to facilitate latrine use.
- $B_{is}$, household demography: 10 demographic controls covering the entire age and sex demographic space in the household.

The counterfactual we aim to calculate is the overall rate of rural open defecation in rural Bangladesh, India, and Nepal, if all households in the region exhibited the same sanitation behaviours of non-Hindu households in predominantly non-Hindu states. We operationalise this by utilising the coefficients on household non-Hindu and state non-Hindu to linearly project rural open defecation using different counterfactual percentages of non-Hindus. The mean rate of rural open defecation is calculated as follows:

(5) $$\bar{OD}=actual\;\bar{OD}+\left(\beta_{1}+\beta_{2}\right)\times \left(counterfactual\;nonHindu\;population-actual\;nonHindu\;population\right).$$

### 4.3. Limitations

These methods may be insufficient for at least three reasons. First, there may be unobservable factors correlated with religion and open defecation. Second, since the fraction of non-Hindus in the state is calculated from DHS data, it may be measured with error, which would attenuate our results. Finally, the DHS question on ‘usual’ defecation behaviour is an improvement on other surveys such as the India Human Development Survey and the Census, which ask about the type of latrine facility owned by the household. However, the DHS question is still not optimal because it does not capture individual variation in latrine use within households. Cross-sectional data show that it is common for individuals within households that have latrines to defecate in the open in this region (Coffey et al., 2014), and that among households that have a latrine, Hindus are much less likely to use it compared to Muslims (Coffey et al., 2017a). This means that our analysis may represent lower bounds on the difference in sanitation behaviour between Hindus and non-Hindus.

## 5. Results

### 5.1. Method one: nonparametric reweighting

This method creates a counterfactual rate of open defecation by reweighting non-Hindus living in predominantly non-Hindu regions to match the distribution on a set of observable characteristics of households living in regions with a higher fraction of Hindus. In order to account for differences in asset wealth, education, and access to electricity and piped water, we split the sample of rural households into bins based on exhibiting these characteristics. In the first scenario, the sample is split into 10 bins based on ownership of nine assets. The second scenario adds binary indicators for household electricity, piped water, and whether any member has more than seven years of education, creating 74 bins.^4^ ^,^ ^5^

To incorporate the effect of social spillovers, we progressively restrict the non-Hindus used for the reweighting based on the fraction of their state that is non-Hindu. In the most restricted estimate, we use non-Hindus living in Mizoram, which is 97 per cent non-Hindu, to project open defecation rates for Hindus and non-Hindus that live in more religiously diverse communities. Supplementary Appendix Table A2 lists state-level averages for religion and open defecation.

**Figure 4.**
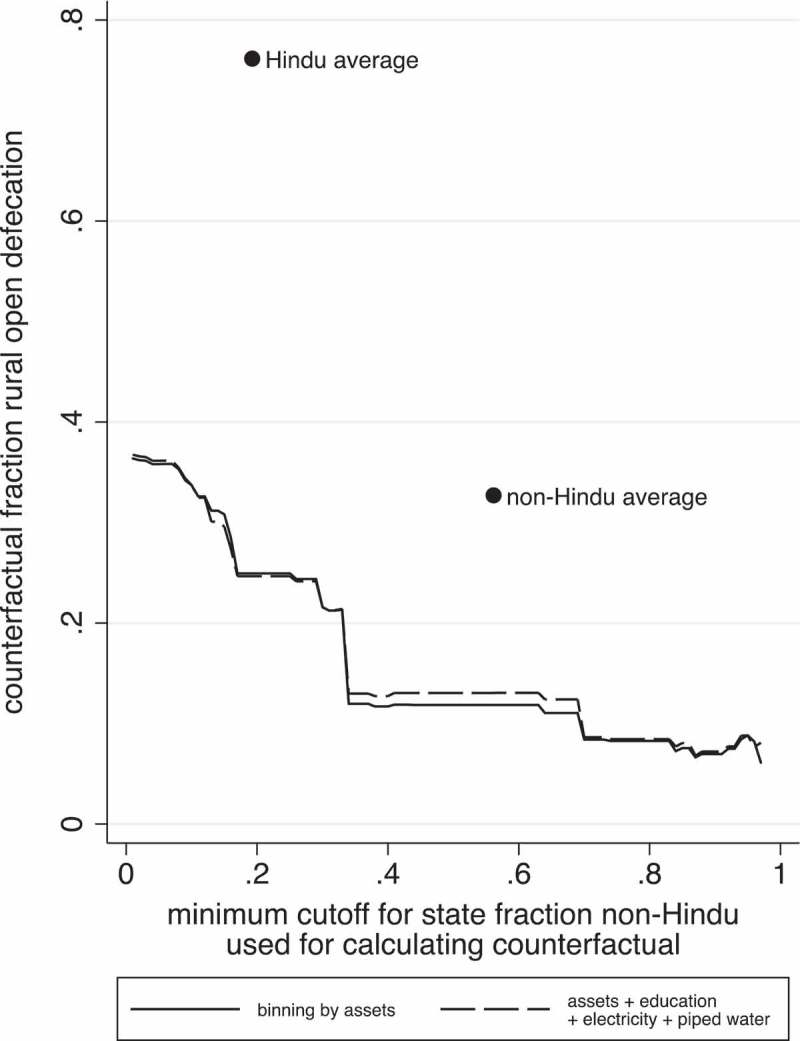
Non-parametric reweighting predicts lower open defecation in rural Bangladesh, India, and Nepal if households in Hindu regions exhibited the sanitation behaviours of non-Hindus in non-Hindu regions. *Notes*: This figure presents counterfactual mean rates of open defecation in rural Bangladesh, India, and Nepal, constructed by reweighting non-Hindu households in regions that have a higher fraction of non-Hindus to match the distribution on a set of observable characteristics of households living in regions that have a higher fraction of Hindus. The solid line bins the sample based on asset count, and the long-dashed line adds indicators for electricity, piped water, and at least one household member having more than seven years of education. Moving from left to right on each line, the sample of non-Hindus used in the reweighting is restricted based on the fraction of non-Hindus in the state in which the household lives. The observed Hindu and non-Hindu sample averages are also displayed for comparison.

Figure 4 uses the nonparametric reweighting method we describe to project the rate of open defecation in rural Bangladesh, India, and Nepal. Each point in this figure represents a projection for the rate of open defecation. Each line represents projections made by splitting the sample into groups based on a cumulative set of observable characteristics. The solid black line is based on splitting the sample into groups based on asset ownership. The long-dashed line splits the sample into groups based on asset ownership, and binary indicators for household electricity, piped water, and at least one person in the household having more than seven years of education. As the graph moves from left to right, we restrict the sample of non-Hindus used for making our projections to those living in more and more non-Hindu states. In the most restricted scenario, the rate of rural open defecation would be between 6 and 8 per cent, compared to the actual level of 65 per cent.

There are two notable aspects in this figure. First, there is not much vertical distance between the projections made using the full set of controls and those made from controlling for assets alone. This suggests that the difference in open defecation rates between Hindus and non-Hindus is not largely explained by educational attainment or infrastructure. Secondly, restricting the sample used for the reweighting to communities with a higher and higher fraction of non-Hindus has a large impact on projected open defecation. In the most restricted scenario, in which we use non-Hindus living in the state with the highest fraction of non-Hindus for the reweighting, the counterfactual open defecation rate is at least 28 percentage points lower than when projections are made using all non-Hindu households for the reweighting. Importantly, in the most restricted scenario, projected open defecation is much lower than even the actual non-Hindu average. This points to the importance of social spillovers.

### 5.2. Method two: linear probability models

Table 4 presents results from linear probability models. Panel (a) displays regression results using six different models, and panel (b) shows projections for rural open defecation using the point estimates from the corresponding six models in panel (a), and different values for the counterfactual fraction of the non-Hindu population.

**Table 4.** Linear probability models predict lower open defecation in rural Bangladesh, India, and Nepal, with a counterfactually higher fraction of non-Hindus

	(1)	(2)	(3)	(4)	(5)	(6)
sample	all models: rural Bangladesh, India, and Nepal					
Panel (a): linear probability models predicting rural open defecation						
non-Hindu	−0.435***		−0.123***	−0.117***	−0.143***	−0.148***
	(0.0143)		(0.0153)	(0.0145)	(0.0133)	(0.0133)
% non-Hindu in state		−0.978***	−0.846***	−0.647***	−0.637***	−0.661***
		(0.0112)	(0.0208)	(0.0218)	(0.0200)	(0.0203)
average rural asset ownership				−0.130***	−0.0551***	−0.0415***
in state				(0.00776)	(0.00738)	(0.00750)
count of nine assets					−0.0626***	−0.0620***
					(0.00162)	(0.00165)
highest level of education					−0.0110***	−0.0129***
					(0.000671)	(0.000699)
household electricity						0.00605
						(0.00731)
household piped water						−0.0930***
						(0.0129)
demographic characteristics						x
N (observations)	77,617	77,617	77,617	77,617	77,514	77,505
Panel (b): predicting overall rural open defecation with a greater fraction of non-Hindus						
100% non-Hindu	34%	−5%	−4%	10%	9%	7%
75% non-Hindu	45%	20%	20%	30%	29%	27%
50% non-Hindu	56%	44%	44%	49%	48%	48%

*Notes*: In Panel (a), standard errors clustered by survey primary sampling unit are in parentheses, and two-sided p-values are displayed as *** p < 0.01, ** p < 0.05, * p < 0.1. Panel (b) displays predictions for overall rural open defecation in Bangladesh, India, and Nepal, calculated using a non-Hindu population counterfactual from the left-hand column and corresponding model coefficients from Panel (a). Thus, each predicted value in Panel (b) is computed as: predicted overall rural open defecation = actual overall open defecation + (non-Hindu coefficient + % non-Hindu in state coefficient) * (counterfactual non-Hindu population – actual non-Hindu population). The actual non-Hindu population rate is 0.291; the actual rural open defecation rate is 0.646.

Column 1 of panel (a) simply shows a significant difference in open defecation rates between Hindu and non-Hindu households; non-Hindus are on average 44 percentage points less likely to defecate in the open than Hindus. Column 2 regresses household open defecation on the fraction of non-Hindu households in the state. The coefficient on the state non-Hindu variable is very large and significant, indicating that state-level norms towards sanitation may be very important for household open defecation. In Column 3, we combine the variables from Columns 1 and 2. The point estimates on both variables attenuate, but they both remain significant and the coefficient on state religion remains very large. To ensure that our state-level religion variable is not merely capturing differences in development across states, we add state-level average rural ownership of nine assets in Column 4. State asset ownership is an important explanatory factor, and does capture some of the variation that was earlier being captured by the state religion variable, but nevertheless, the point estimate on state religion remains large and significant.

In Column 5, we begin to add household-level covariates. We first add asset ownership and educational attainment. The model in Column 6 adds electricity, piped water supply, and 10 age-sex demographic controls covering the entire demographic space of the household separately for males and females. These additions change the point estimates on the religion variables very little. The model with the full set of controls indicates that a non-Hindu household in a fully non-Hindu state is, on average, 81 percentage points less likely to defecate in the open than a Hindu household in a fully Hindu state.^6^

Several important conclusions arise from this table. First, although household religion is important for explaining some variation in open defecation, the religious context in which a household lives appears to be more important. This implies that religion’s effect on sanitation behaviour is mediated through social norms more than household-level differences. Another important conclusion is the relative unimportance of wealth, education, and household infrastructure, for latrine use in this region. The point estimates in Column 6 suggest that, controlling for other variables, households in the 75th percentile of asset ownership, who own four assets, are on average 19 percentage points less likely to defecate in the open than households in the 25th percentile, who own one asset. Similarly, households with at least one member who studied for 10 years (75th percentile) are only eight percentage points less likely to defecate in the open, on average, than households with at least one member who studied for four years (25th percentile). The point estimates on electricity and piped water access are similarly small. In comparison, Hindu families living in the state with the highest representation of non-Hindus are at least 59 percentage points less likely to defecate in the open, on average, than Hindu families living in the state with the lowest representation of non-Hindus. These results provide evidence that wealth, education, and infrastructure, factors that are widely recognised in the literature to be associated with latrine use, are relatively less important in South Asia in comparison to social factors like religious beliefs.

Panel (b) projects open defecation rates in rural Bangladesh, India, and Nepal, using the models from panel (a) and different values for the counterfactual fraction of the non-Hindu population. The first row in panel (b) presents projections if the counterfactual non-Hindu population were 100 per cent, and the following rows present projections assigning lower percentages to the representation of non-Hindus.^7^ The models in Columns 2 and 3 lead to projections of rural open defecation that are negative because of the unrealistic assumption of linearity implicit in linear probability models. Using models with controls, the projected rate of rural open defecation if the non-Hindu population were 100 per cent is between 7 and 10 per cent. Notably, these projections are similar to those from nonparametric reweighting.

## 6. Discussion

This paper investigated differences in rates of open defecation among countries in South Asia, noting that rural places with a higher fraction of Hindu households also have a higher fraction of households defecating in the open, a relationship that is not explained by differences in wealth. We find evidence of social spillovers: Hindus and non-Hindus alike are more likely to defecate in the open when they live around other Hindus, and less likely to when they live around other non-Hindus. Applying demographic reweighting techniques to data from rural Bangladesh, India, and Nepal, we project a rate of rural open defecation if rural households counterfactually demonstrated the same behaviour as non-Hindus living in predominantly non-Hindu areas. Our methods take advantage of both individual differences between households, and aggregate differences between households living in states with varying religious representation. In practice, we exploit the behaviour of households living in Bangladesh and certain states of India that are majority non-Hindu to make our projections. We estimate that rural open defecation in Bangladesh, India, and Nepal would have been between 6 and 8 per cent, compared to the actual 65 per cent, if rural households in the region demonstrated the same sanitation behaviours as non-Hindu households living in the most non-Hindu areas.

Since India and Nepal represent a large fraction of the world’s open defecation, the number of people that still defecate in the open worldwide would be roughly halved if households living in predominantly Hindu regions in rural India and Nepal altered their behaviour to reflect that of non-Hindus in the most non-Hindu regions. The association between sanitation and religion also more than accounts for the difference in rural open defecation rates between South Asia and sub-Saharan Africa, where 40 per cent of households defecated in the open in 2005. South Asia’s rural open defecation rates are expected to be even lower than sub-Saharan Africa’s because South Asians are richer on average.

The results should be interpreted in the context of other factors that are linked to latrine adoption: wealth, education, electricity coverage, and piped water access. Although these factors are significantly associated with open defecation in the region, the magnitudes of these associations are small relative to that of religion and open defecation. This finding aligns with recent evidence that economic, education, demographic, and home improvement changes only weakly explain latrine adoption in rural India between 2005 and 2012 (Coffey, Spears, & Vyas, 2017b).

The results of this paper are best understood in the context of sanitation policy in India and Nepal, the two South Asian countries in which open defecation is still very prevalent. Nepal’s government initiated sanitation programming in 1994, under which some projects provided subsidies for toilet construction. Since India’s rural sanitation policy was initiated three decades ago, it has focused primarily on latrine construction. The effects of these programmes have been limited: open defecation has been decreasing at an annual rate of roughly two percentage points in rural Nepal, and one percentage point in rural India. Moreover, research on latrine use in rural north India and the Nepali terai has shown that it is common for individuals to defecate in the open even if they have a latrine (Coffey et al., 2014, 2017a).

The failure of these policies to accelerate the reduction of open defecation in these countries signal that affordability is not the binding constraint. The findings of this study suggest, instead, that social norms and beliefs rooted in purity and pollution are. In this context, policies that focus on making latrines and pit emptying more socially acceptable, and that address communities, rather than individual households, may be more likely to succeed in changing social norms around sanitation and persuading rural households to use the kinds of latrines that most can already afford. Unfortunately, it is not yet clear what sorts of interventions could accomplish this, because to our knowledge, no intervention studies that specifically address issues related to purity and pollution have been completed. However, instances of broad shifts in social attitudes in other contexts on other issues are encouraging.^8^ Community-based interventions that address sanitation behaviour in India represent an important new avenue for research in South Asia.

The toll that open defecation takes on health and human development indicates that eliminating open defecation should be a policy priority. As India gets richer, there is reason to believe that sanitation will improve, not only because more households will be able to afford the kinds of latrines that they prefer, but because as more households switch to latrine use, social norms around sanitation will change, affecting rich and poor alike. However, economic development will take time. In the meantime, interventions addressing social norms and beliefs related to latrines and pit emptying would likely speed latrine adoption a great deal, and contribute to a better environment for India’s children.

## Notes

1. Literature in the medical and epidemiological fields link poor sanitation to poor nutritional outcomes and child height (Pickering, Djebbari, Lopez, Coulibaly, & Alzua, 2015) through diarrhoea (Checkley et al., 2004), intestinal parasites (Esrey, Potash, Roberts, & Shiff, 1991), and environmental enteropathy (Humphrey, 2009). A meta-analysis conducted by Gertler et al. (2015) uses experimentally-induced variation in open defecation to identify a causal effect of open defecation on child height. Within India, Spears and Lamba (2015) find an effect of a nationwide sanitation campaign in India on the cognitive development of six-year-olds, and Lawson and Spears (2015) links open defecation to adult wages.

2.
Figure 2 shows smaller differences in open defecation between Hindus and non-Hindus for higher levels of asset wealth in Bangladesh and India. It is important to note, however, that the number of observations used to construct mean open defecation rates for higher asset ownership households is much smaller than for lower asset ownership households. See Supplementary Materials Figure A1 for the cumulative distributions of asset ownership.

3. This method is also employed by Coffey (2015), Spears (2013), and Geruso (2012).

4. The number of groups is less than 80 because some combinations of assets, education, electricity, and piped water do not have any households associated with them.

5. Results from binning households by assets and education only, and by assets, education and electricity only, are very similar to the results from the scenarios we display, and are thus omitted for simplicity.

6. The association between social norms, indicated by the fraction of non-Hindus in the state, and open defecation is not statistically different between Hindus and non-Hindus; when controlling for state wealth, household wealth, household education, and household infrastructure, the interaction between household non-Hindu and fraction non-Hindu in the state is insignificant.

7. Since the actual representation of non-Hindus in the region is 29 per cent, projections of open defecation assigning a representation of non-Hindus of 25 per cent or lower are not shown.

8. For example, attitudes on same-sex marriage have changed considerably over time in the United States (Baunach, 2012).
