# Supplementary material for: Sanitation and Religion in South Asia: What Accounts for Differences across Countries?
Source: J Dev Stud. 2018 May 18;54(11):2119–35. doi: 10.1080/00220388.2018.1469742 (PMC6183933; doi:10.1080/00220388.2018.1469742)
Supplement: Supplementary Materials [file FJDS_A_1469742_SM8595.pdf]

Supplementary Appendix

A1. Variable construction

In Bangladesh, 89.8 per cent of households are Muslim and 0.5 per cent are Christian or Buddhist, while Hindus comprise the largest minority. Hindus represent a majority in India and Nepal. In India, Muslims are the largest minority (11.5%), followed by Christians (2.3%), Sikhs (1.6%), Buddhists (0.8%), and Jains (0.07%). In Nepal, Buddhists are the largest minority (8.7%), followed by Muslims (3.4%), Kirati (1.7%), and Christians (1.5%).

In India, the DHS collected the religion of the household head. For Bangladesh and Nepal, however, the women interviewed in the household were individually asked their own religion. In 2 per cent of total households in Nepal and 0.1 per cent of total households in Bangladesh, the two or more women interviewed within the household responded that they adhere to different religions. For these households, we assign the religion that occurs most often among the women interviewed in the household. We drop households if no single religion occurs most often, which is the case for roughly half the inter-faith households in Nepal and most of the inter-faith households in Bangladesh. We also drop households in which no women were interviewed because data on religion was not recorded for these households.

Figure A1: Cumulative distribution of rural asset ownership in rural Bangladesh, India, and Nepal

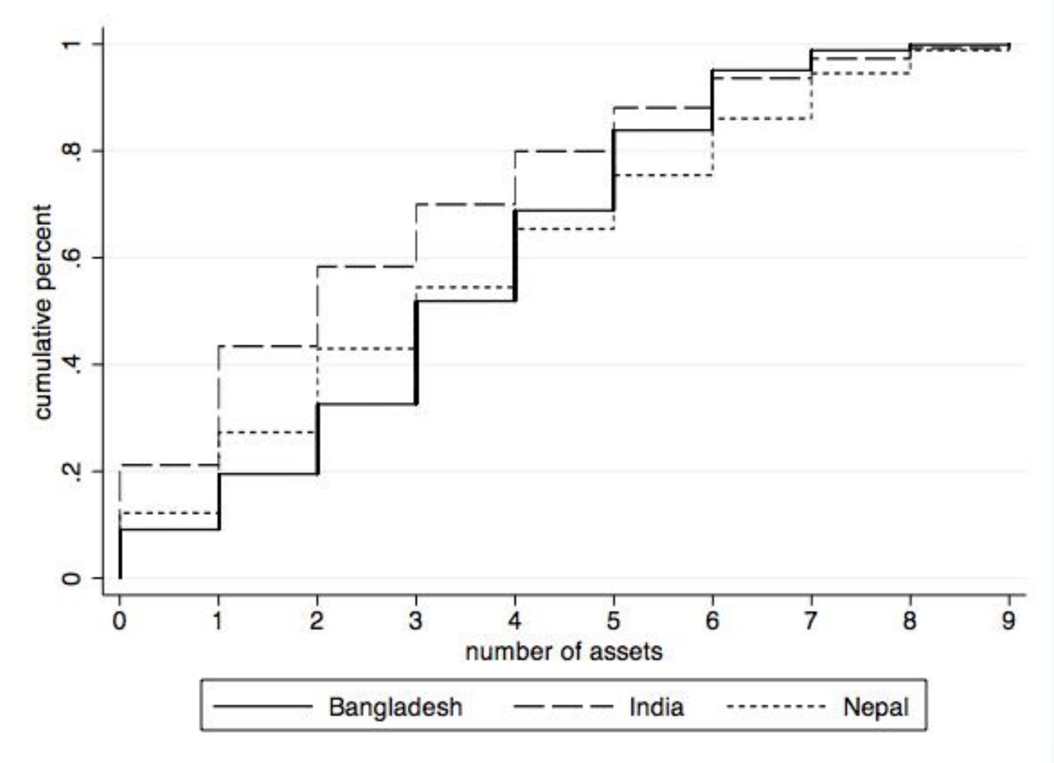

Figure presents the cumulative distribution functions of rural asset ownership, separately for Bangladesh, India, and Nepal.

Figure A2: Differences in sanitation behaviour within and between Hindus and non-Hindus in rural Bangladesh, India, and Nepal

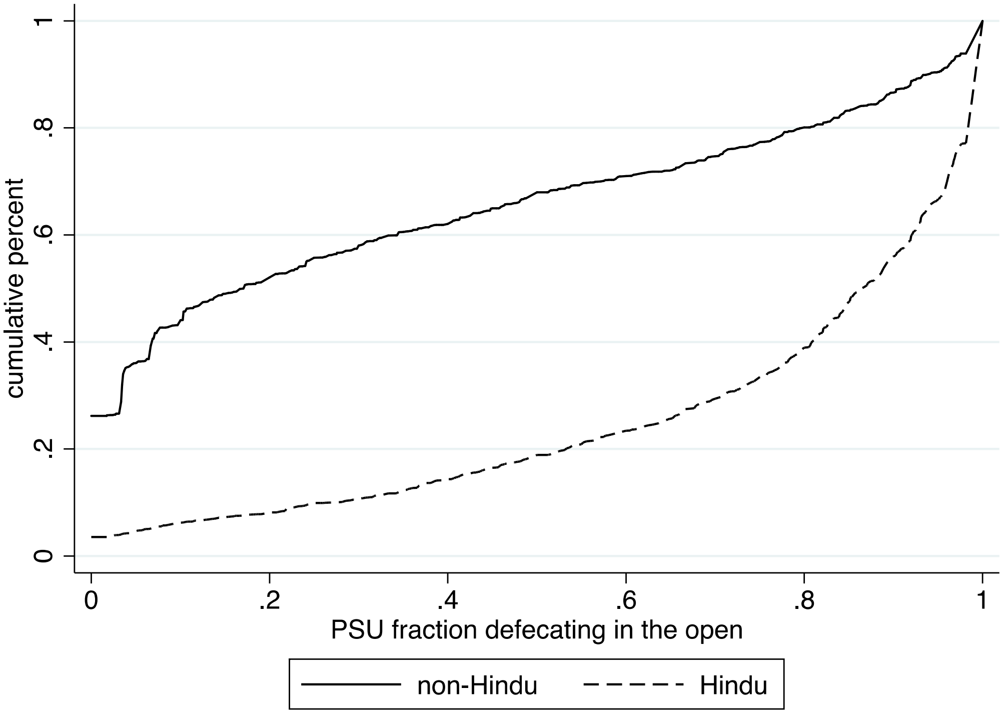

Figure presents the cumulative distribution functions of the PSU fraction of households defecating in the open, separately for Hindus and non-Hindus.

Table A1: Description of DHS surveys and data availability

country	survey year	DHS sample size	Data availability					
			sanitation	religion	electricity	piped water	literacy	assets
Afghanistan	2010	22,351	x		x	x		x
Bangladesh	2011	17,141	x	x	x	x	x	x
Bhutan	No available DHS							
India	2005-2006	109,041	x	x	x	x	x	x
Maldives	2009	6,443	x		x	x		x
Nepal	2011	10,826	x	x	x	x	x	x
Pakistan	2012-2013	12,943	x		x	x	x	x
Sri Lanka	Last available data from 1987							

Table A2: Open defecation and Hinduism in divisions, states, and regions, in Bangladesh, India, and Nepal, respectively

country	division/state/region	total per cent open defecation	total per cent Hindu	rural per cent open defecation
(1)	(2)	(3)	(4)	(5)
Bangladesh	Rangpur	12.4%	15.9%	14.4%
Bangladesh	Rajshahi	3.8%	5.8%	5.1%
Bangladesh	Dhaka	3.8%	4.6%	6.4%
Bangladesh	Sylhet	3.3%	14.4%	4.8%
Bangladesh	Chittagong	2.0%	8.2%	2.9%
Bangladesh	Khulna	1.8%	10.6%	2.5%
Bangladesh	Barisal	1.6%	7.9%	1.9%
India	Chhattisgarh	74.9%	93.9%	94.4%
India	Orissa	74.3%	96.3%	88.6%
India	Jharkhand	70.0%	74.2%	95.0%
India	Rajasthan	64.9%	87.7%	91.6%
India	Bihar	61.7%	82.6%	83.8%
India	Karnataka	54.7%	85.5%	78.1%
India	Uttar Pradesh	54.5%	78.9%	84.0%
India	Madhya Pradesh	50.5%	88.3%	90.4%
India	Haryana	49.5%	88.0%	64.5%
India	Tamil Nadu	47.0%	88.3%	83.0%
India	Gujarat	45.7%	90.5%	69.8%
India	Himachal Pradesh	43.1%	95.3%	59.9%
India	Uttaranchal	41.5%	86.9%	57.7%
India	Jammu and Kashmir	36.8%	37.9%	49.1%
India	Andhra Pradesh	34.5%	77.9%	73.1%
India	Punjab	29.5%	42.9%	44.0%
India	Maharashtra	29.4%	75.8%	79.5%
India	West Bengal	26.6%	73.5%	55.1%
India	Goa	26.1%	65.2%	38.2%
India	Meghalaya	22.3%	19.5%	38.3%
India	Assam	19.8%	72.2%	28.9%
India	Arunachal Pradesh	18.9%	34.1%	25.4%
India	Nagaland	10.3%	15.9%	19.5%
India	Delhi	10.2%	85.4%	28.0%
India	Sikkim	8.7%	59.0%	13.7%
India	Kerala	3.8%	59.5%	4.9%
India	Manipur	3.7%	56.6%	6.4%
India	Tripura	2.9%	88.9%	3.8%
India	Mizoram	2.0%	3.0%	4.2%
Nepal	Mountain	35.6%	62.2%	37.8%
Nepal	Terai	33.6%	74.3%	45.3%
Nepal	Hill	29.2%	68.6%	37.4%

Means calculated using sampling weights.
